# The antifibrotic and anti-inflammatory effects of FZHY prescription on the kidney in rats after unilateral ureteral obstruction

**DOI:** 10.1590/acb371003

**Published:** 2023-01-06

**Authors:** Ziwei Chen, Shaobo Wu, Yu Zeng, Xueying Li, Mengping Wang, Zejun Chen, Ming Chen

**Affiliations:** 1 M.M. Chengdu University of Traditional Chinese Medicine – Department of Nephrology – Affiliated Integrated TCM and Western Medicine Hospital of Chengdu – Chengdu Integrated TCM and Western Medicine Hospital – Chengdu First People’s Hospital – Chengdu, China.; 2 M.M. Chengdu University of Traditional Chinese Medicine – Department of Nephrology – Hospital of Chengdu – Chengdu, China.; 3 B.S. Chengdu University of Traditional Chinese Medicine – Department of Clinical Laboratory – Hospital of Chengdu – Chengdu, China.; 4 M.D. Chengdu University of Traditional Chinese Medicine – Department of Nephrology – Affiliated Integrated TCM and Western Medicine Hospital of Chengdu – Chengdu Integrated TCM and Western Medicine Hospital – Chengdu First People’s Hospital – Chengdu, China.

**Keywords:** FZHY, Chronic kidney disease, Fibrosis, Inflammation, CD4 Antigens, CD8 Antigens

## Abstract

**Purpose:**

To explore the potential impact of traditional Chinese herb FuZhengHuaYuJiangZhuTongLuo recipe (FZHY) on renal interstitial fibrosis (RIF) in chronic kidney disease (CKD) at cellular and molecular levels.

**Methods:**

Unilateral ureteral obstruction (UUO) rats were established as the RIF model *in vivo*. The rats were given intragastric administration with FZHY once a day for consecutive 7, 14 and 21 days, respectively. The renal function parameters and inflammation indicators in kidney tissues were measured using enzyme-linked immunosorbent assay, the CD4^+^/CD8^+^ T cells in peripheral blood was detected using flow cytometry, the renal fibrosis degree was estimated using Masson’s staining, and the fibrosis-related genes’ expression was detected using quantitative polymerase chain reaction, western blotting, and immunohistochemistry analyses.

**Results:**

FZHY prescription reduced the serum creatinine and blood urea nitrogen, decreased the levels of c-reactive protein, interleukin-1, interleukin-6 and tumor necrosis factor-α in kidney tissues, and increased the ratio of CD4^+^/CD8^+^ T cells in peripheral blood. FZHY prescription suppressed the renal tissue fibrosis and reduced the levels of laminin, fibronectin, collagen I and collagen III.

**Conclusions:**

FZHY prescription suppressed the renal fibrosis and improved the condition of “Healthy Qi Deficiency and Evil Qi Excess” in rats with UUO, which may provide an effective method for CKD treatment.

## Introduction

Chronic kidney diseases (CKD), affecting around 18% of the worldwide population, cause high morbidity and mortality and increasingly attract researchers’ attentions worldwide^1^. Renal interstitial fibrosis (RIF) is the main pathological feature occurring in the middle and late stages of CKD, and it is the most common pathological process in the progression of CKD to end-stage renal failure (ESRD)^2^. The main characteristics of RIF include renal tubule atrophy or expansion, epithelial cell shedding, interstitial inflammatory cell infiltration and massive deposition of extracellular matrix (ECM)^3^. The main anti-fibrosis treatment strategies currently available are dialysis or kidney transplantation. However, the increased mortality and recurrence rate confirm that these treatments are not sufficient to effectively moderate the progression of CKD to ESRD^4^. Therefore, understanding the mechanism of RIF is of great significance to the prognosis of RIF.

Immune mechanisms are gradually considered as a prerequisite for the progression of chronic disease, with systemic inflammation and immune deficiency prevalent in patients with CKD or in animal models^5^,^6^. Phenotypic changes and dysfunction of T cells are associated with the progression of CKD^7^. Long-term amplified inflammatory signals can change the function of T cells, whether T-lymphocytes including CD4^+^ and CD8^+^ subsets are characterized by immune damage or loss of effector functions^8^,^9^. Indeed, a growing number of studies suggest that lymphocytopenia occurs in patients with CKD or ERSD, in part as immune dysfunction induced by T cell-mediated changes in the absolute number of CD4^+^ and CD8^+^ T cells^10^,^11^, even the absolute number of CD4^+^ and CD8^+^ in renal fibrosis^11^-^13^. However, the changes in the relative number of CD4^+^/CD8^+^ T cells in RIF have not been specifically studied.

In China, traditional Chinese medicine (TCM) has a long history of treating kidney disease and is still used as an alternative therapy for renal disorders. TCM has its unique advantages in improving the quality of life and long-term survival of patients. Clinical trials have validated that numbers of Chinese herbal formulas are effective for RIF, with active ingredients, such as tripterygium glycosides, resveratrol, and astragaloside. FuZhengHuaYuJiangZhuTongLuo recipe (FZHY) prescription is a traditional Chinese herb that significantly ameliorates the chronic renal failure through anti-inflammatory and immunomodulatory effects in 5/6 nephrectomy model^14^. It contains nine traditional Chinese herbs including raw astragalus, *Rehmannia glutinosa*, salvia, safflower, wine leeches, soil beetle, wine scutellaria, wine rhubarb, and raw licorice. The co-administration of wine rhubarb and raw astragalus is used to regulate and harmonize the spleen and stomach, and the raw astragalus strengthens the spleen and reinforces healthy qi, and the wine rhubarb unblocks the bowel and exorcises the pathogenic factors. The co-administration of raw astragalus and *Rehmannia glutinosa* is performed to achieve the purpose of tonifying the spleen and kidney, raw astragalus focuses on strengthening the spleen, while *Rehmannia* plays key function on tonifying the kidney. The co-administration of wine rhubarb and raw licorice is used to rehabilitate the spleen-stomach function, wine rhubarb removes damp-heat stasis toxin in intestine, and the function of raw licorice is detoxication. The co-administration of the cold salvia and the warm safflower is used to circulate blood, eliminate stasis, and unblock meridians, which rehabilitates the blood circulating in the heart, contained in the spleen and stored in the liver. The co-administration of the wine leeches and the soil beetle is used for blood stasis-removing and meridian-collateral-dredging. In addition, the wine scutellaria reduces fire and dry dampness, and it does not damage the spleen yang. Therefore, FZHY has the function of reinforcing the spleen and benefiting the kidney, invigorating qi and strengthen the body, and unblocking the bowel and transform turbidity.

However, the potential anti-fibrosis effect of FZHY has not been reported. Unilateral ureteral obstruction (UUO) in rats is considered the most common animal models of chronic kidney pathologies, including interstitial fibrosis^15^. Therefore, in this study, we adopted a UUO rat model of RIF and tried to examine the possible protective effect and mechanism of FZHY on the pathology of RIF *in vivo*.

## Methods

### Animals and unilateral ureteral obstruction model

Male Sprague-Dawley rats (7-8 weeks) weighing 240 to 280 g were purchased from Laboratory Animal Business Department, Shanghai Institute of Planned Parenthood Research (Shanghai, China). All rats were given adaptive feeding for one week and randomly divided into four groups: Sham (n = 12), UUO (n = 12), UUO + FZHY (n = 12), and UUO + AST-120 (n = 12) – AST-120 is a type of oral spherical activated carbon particles (commercial name KREMEZIN^®^) that adsorb uremic toxins and their precursors within the gastrointestinal tract and has been proven to treat CKD effectively and, therefore, serves as a positive control. The UUO model was estimated as follows. Shortly, all rats were anesthetized by intraperitoneal injection of sodium pentobarbital. The abdominal cavity was opened through the left abdominal incision. The left ureter was bluntly separated and double-ligated with 4-0 sutures in the middle and upper 1/3, and the abdominal cavity was closed by layered suture. The rats in sham group underwent a similar operation, but the ureter was not ligated. The rats were used in accordance with the National Institutes of Health Guidelines for the Use of Laboratory Animals, and this study was approved by the Ethics Committee of our institution (approval no.: 2021DL-016). FZHY is a Chinese medicine developed by us, and AST-120 has been shown to improve the progression of CKD as a positive control^16^. The dose of FZHY for each rat was 4.92 g/kg/d. The dose of AST-120 for each rat was 4 g/kg/d. Rats were given intragastric administration once a day for 7, 14 and 21 days, respectively. Afterwards, seven rats were randomly selected from each group and sacrificed. Blood and kidney tissues were collected for subsequent experiments.

### Masson’s trichrome staining and immunohistochemical analysis

The degree of renal tissue injury was evaluated by Masson’s trichrome staining. The kidney tissues were fixed in 4% paraformaldehyde, embedded in paraffin, and sliced into 4-μm paraffin sections. Then, sections were treated in xylene, dehydrated with graded ethanol, and stained with Masson (Sigma-Aldrich; Merck KGaA). After staining, the sections were dehydrated with 70 and 90% ethanol. Six fields of view were randomly selected and observed with an optical microscope (Olympus, Tokyo, Japan).

For immunohistochemistry (IHC) analysis, the renal tissues were incubated with the primary antibodies including anti-LN (CY6617, 1:100, Abways), anti-FN (CY5621, 1:100, Abways), anti-Col-I (AF0127, 1:200, Affinity), anti-Col-III (AF0136, 1:100, Affinity) overnight at 4 °C, respectively, and then further incubated with an anti-rabbit secondary antibody (ab150077, Abcam) for 2 h at room temperature. Finally, a representative area containing immunostained tissue was captured with a microscope (Olympus, Tokyo, Japan).

### Flow cytometry: measurements of CD4^+^/CD8^+^ T cell ratio

Rat PBMC was obtained and isolated from rat peripheral blood using Ficoll gradient. The red blood cells were lysed using the lysis buffer (C3702, Biolegend), and then harvested and stained with CD4-FITC anti-rat CD4 (Biolegend) and CD8-PE (200607, Biolegend). Then, the CD4^+^/CD8^+^ T cell ratio was calculated. All fluorescent samples were analyzed with a FACS Canto II flow cytometer (BD Biosciences).

### Enzyme-linked immunosorbent assay

Blood samples from rats were used to detect biochemical markers. Serum samples were collected by centrifugation at 3,000 rpm for 20 min, and serum creatinine (SCR) (ml058879) and blood urea nitrogen (BUN) (ml730662) were measured by enzyme-linked immunosorbent assay (ELISA) kits (Shanghai Enzyme-linked Biotechnology Co., Ltd.), respectively. Kidney tissue samples from rats were used to detect inflammatory cytokines. The C-reactive protein (CRP, ml038253), tumor necrosis factor-α (TNF-α, ml002859), interleukin-6 (IL-6, ml102828) and interleukin-1 (IL-1, ml037373) were measured by ELISA kits (Shanghai Enzyme-linked Biotechnology Co., Ltd.).

### Statistical analysis

Statistical analysis was performed using GraphPad Prism 8.0 (GraphPad Software, San Diego, United States of America) and presented with mean ± standard deviation (SD). When appropriate, one-way analysis of variance (ANOVA) (followed by Tukey’s test) or Brown-Forsythe and Welch ANOVA was used to detect the statistical differences among groups. The significance was defined by *P* < 0.05, 0.01 and marked as *, **, #, ##, D, and DD.

## Results

### FZHY treatment improved kidney function in unilateral ureteral obstruction model rats

**Figure 1:**
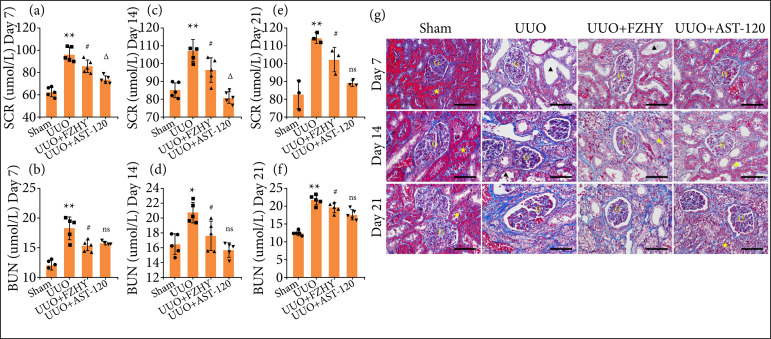
FZHY alleviated the renal injury in UUO rats. The amount of serum creatinine (SCR) and blood urea nitrogen (BUN) for rats in sham, UUO, UUO + FZHY and UUO + AST-120 groups on (**a**, **b**; n = 5) day 7, (**c**, **d**; n = 5) day 14, and (**e**, n = 3; f, n = 5) day 21. **(g)** The renal fibrosis assessment was detected by the Masson’s trichrome staining (400 × magnification) on day 7, day 14 and day 21, separately.

FZHY: FuZhengHuaYuJiangZhuTongLuo recipe; UUO: unilateral ureteral obstruction; *P < 0.05, **P < 0.01 UUO group *vs*. sham group; ^#^ *P* < 0.05, ^##^ *P* < 0.01, UUO + FZHY group *vs*. UUO group; ^D^ *P* < 0.05; ^DD^ *P* < 0.01; ns: not significant; UUO + AST-120 group *vs*. UUO + FZHY group; G: glomerulus; ▲: dilatated tubule; ★: tubule. Scale bar: 50 μm.

Compared with sham group, the ELISA results showed that the UUO group significantly increased SCR and BUN levels on days 7, 14 and 21 in UUO model rats, while FZHY treatment effectively reduced SCR and BUN levels on days 7, 14 and 21 in UUO model rats. Further, FZHY treatment slightly but significantly (*P* < 0.05) alleviated the levels of SCR and BUN levels in UUO kidneys, although the reducing effect didn’t reach the extent as AST-120 caused on days 7 and 14 (Figs. 1a-1d). FZHY treatment alleviated SCR and BUN levels in UUO kidneys on day 21, showing no statistically significant difference from AST-120 (Figs. 1e-1f). In addition, compared with sham group, the UUO group showed significant renal tissue structure injury and renal fibrosis accompanied by a large amount of collagen deposition on days 7, 14 and 21. Instead, FZHY treatment markedly improved the damage and fibrosis, similar to the effect of AST-120 (Fig. 1g).

### FZHY treatment attenuated the inflammation in kidney of unilateral ureteral obstruction model rats

**Figure 2:**
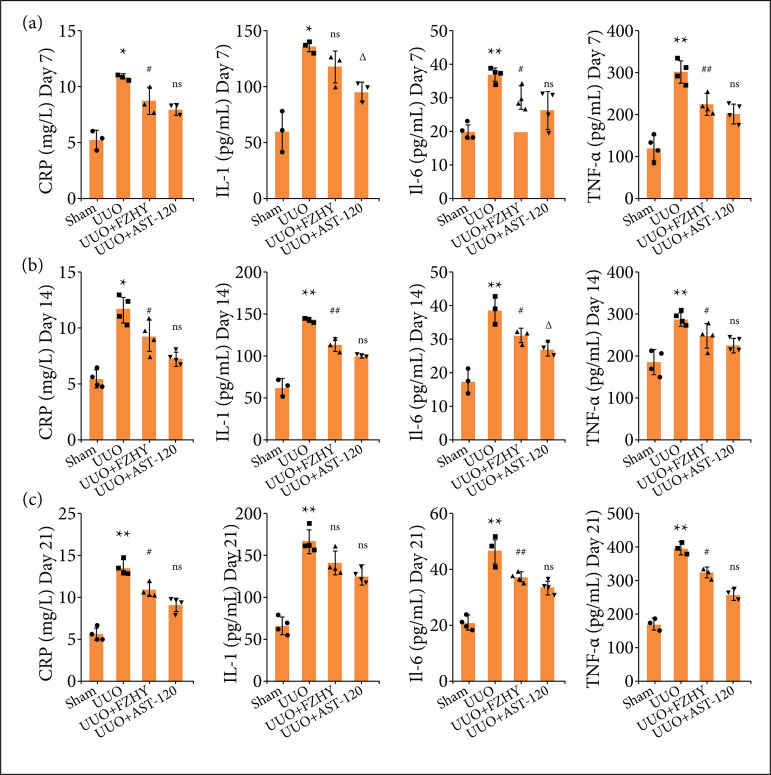
FZHY inhibited the inflammatory response in UUO rats. The inflammation markers such as CRP, IL-1, IL-6 and TNF-α were assessed in sham group, UUO model group, UUO + FZHY and UUO + AST-120 groups on (**a**; CRP, n = 3; IL-1, n = 3; IL-6, n = 4; TNF-α, n = 4) day 7, (**b**; CRP, n = 4; IL-1, n = 3; IL-6, n = 3; TNF-α, n = 4) day 14, and (**c**; CRP, n = 4; IL-1, n = 4; IL-6, n = 4; TNF-α, n = 3) day 21.

FZHY: FuZhengHuaYuJiangZhuTongLuo recipe; UUO: unilateral ureteral obstruction; CRP: C-reactive protein; IL-1: interleukin-6; IL-6: interleukin-6; TNF-α: tumor necrosis factor-α; *P < 0.05; **P < 0.01 UUO group *vs*. sham group; ^#^ *P* < 0.05; ^##^ *P* < 0.01; NS: not significant; UUO + FZHY group *vs*. UUO group; ^D^ *P* < 0.05; ^DD^ *P* < 0.01; ns: not significant; UUO + AST-120 group *vs*. UUO+FZHY group.

Compared with sham group, the ELISA results showed that the UUO group had a significant increase in the secretion of inflammatory factors CRP, IL-1, IL-6 and TNF-α in renal tissues on days 7, 14 and 21 (Figs. 2a-2c). However, compared with UUO group, FZHY treatment effectively reduced the production of CRP, IL-1, IL-6 and TNF-α on the 14th day and on the 21st day, showing a slight or no significant differences from those in the AST-120 group, respectively (Figs. 2b and 2c). In addition, FZHY treatment significantly reduced CRP, TNF-α and IL-6 levels on day 7, but it did not significantly affect IL-1 levels (Fig. 2a). In general, both FZHY and AST-120 were able to inhibit the increase of inflammatory factors caused by UUO, and as time went on, FZHY might have a better efficacy, evidenced by no significant differences in all inflammatory factors between FZHY and AST-120 treatments on day 21.

### FZHY treatment increased the ratio of CD4^+^/CD8^+^ T cell ratio in unilateral ureteral obstruction model rats

**Figure 3:**
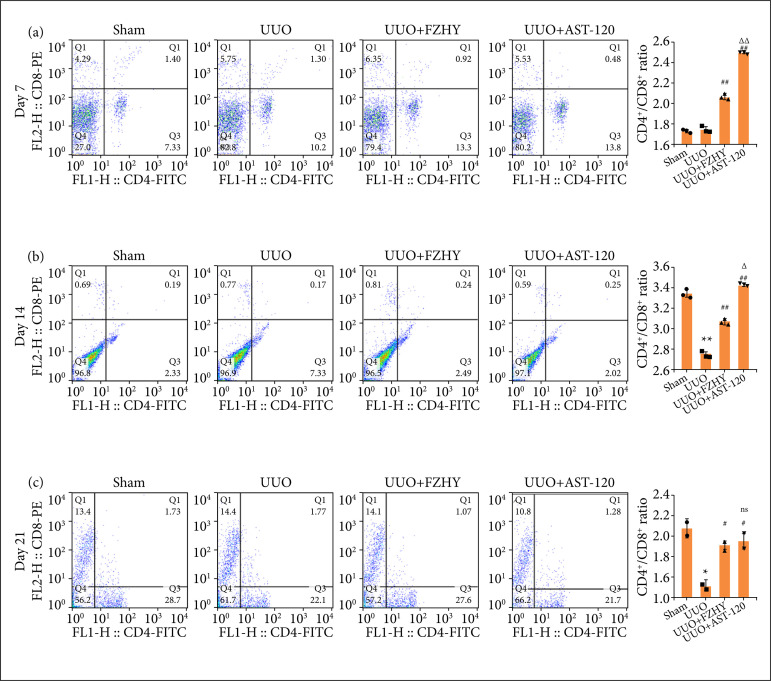
FZHY changed the function of the immune system in UUO rats. The ratio of CD4^+^/CD8^+^ T cells was determined in sham group, UUO model group and UUO + FZHY group on (**a**, n = 3) day 7, (**b**, n = 3) day 14, and (**c**, n = 2) day 21, and the histogram and statistical test for the ratio of CD4^+^/CD8^+^ were performed by GraphPad software on day 7, day 14, and day 21, respectively.

FZHY: FuZhengHuaYuJiangZhuTongLuo recipe; UUO: unilateral ureteral obstruction; *P < 0.05; **P < 0.01; ns: not significant.

Compared with sham group, the flow cytometry results revealed that the ratio of CD4^+^/CD8^+^ T cells was significantly reduced in peripheral blood of the UUO group on days 14 and 21, but not on day 7. FZHY or AST-120 treatment significantly increased the proportion of CD4^+^/CD8^+^ T cells in the rats after UUO on days 7, 14 and 21 (Figs. 3 a-3c). Comparing the two types of treatment, AST-120 had seemingly better effects than FZHY, but they had a very similar result on day 21 (Fig. 3c).

### FZHY treatment protected renal against fibrosis in unilateral ureteral obstruction model rats

**Figure 4:**
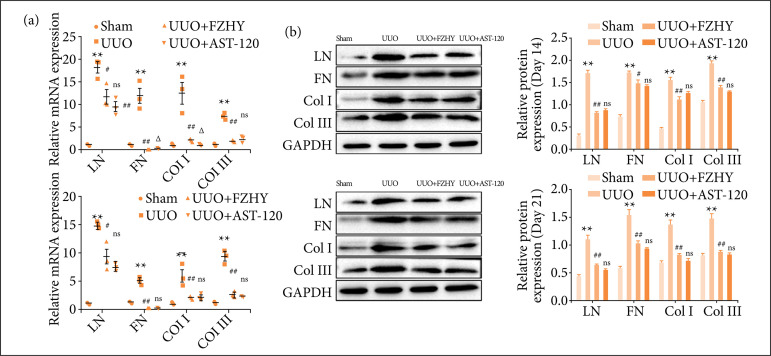
FZHY reduced the mRNA and protein levels of the renal interstitial fibrosis markers in UUO rats. The (**a**, n = 3) mRNA and (**b**, n = 3) protein levels of the renal interstitial fibrosis markers LN, FN, Col-I and Col-III were evaluated in sham, UUO, UUO + FZHY and UUO + AST-120 groups on day 14 and day 21.

FZHY: FuZhengHuaYuJiangZhuTongLuo recipe; UUO: unilateral ureteral obstruction; **P < 0.01, UUO group *vs*. sham group; ^#^ *P* < 0.05; ^##^ *P* < 0.01, UUO+FZHY group *vs*. UUO group; D P < 0.05, UUO+AST-120 group *vs*. UUO+FZHY group; ns: not significant.

**Figure 5:**
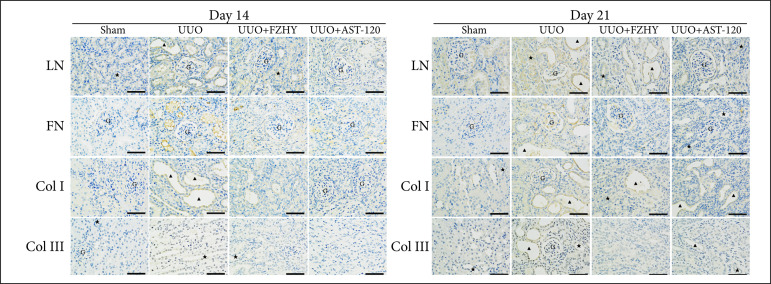
The renal interstitial fibrosis markers in UUO rats were detected by immunohistochemical staining. The immunohistochemical staining of LN, FN, Col-I and Col-III (×400) were detected in sham group (n = 3), UUO model group (n = 3), FZHY-treated group (n = 3) and AST-120-treated group (n = 3) on day 14 and day 21.

FZHY: FuZhengHuaYuJiangZhuTongLuo recipe; UUO: unilateral ureteral obstruction; G: glomerulus; : dilatated tubule; : tubule. Scale bar: 50 μm.

Both mRNA and protein levels of renal fibrosis-related proteins FN, LN, Col-I and Col-III were significantly increased after UUO, but, compared with UUO group, they were significantly decreased after FZHY or AST-120 treatment on the 14th day and on the 21st day (Figs. 4a and 4b). The corresponding results of IHC showed that, compared with sham group, the UUO group damaged the renal tissue structure, promoted the dilatation of renal tubules and elevated the levels of renal fibrosis-related proteins LN (in tubules and glomeruli), FN (in tubules and interstitium), Col-I (in tubules and interstitium) and Col-III (in tubules and glomeruli) in the kidney tissue on days 14 and 21. In addition, compared with UUO group, FZHY treatment significantly decreased the structural damage and reduced their levels in the kidney tissue (Fig. 5).

## Discussion

In previous studies, we obtained the core ingredients of FZHY for the treatment of chronic renal failure (chronic kidney disease), such as torachrysone-8-O-beta-D-(6’-oxayl)-glucoside, hederagenin, stigmasterol and isotanshinone II, by network pharmacology (to be published, Suppl. Table 1). However, the curative effect and mechanism of FZHY on CKD need to be further verified and explored. UUO model is a classic RIF disease model, with both immune abnormalities and microinflammation, which conforms to the condition of “Healthy Qi Deficiency and Evil Qi Excess”.

In this study, we estimated an *in vivo* model of UUO and investigated the effects of FZHY on the treatment of RIF within a specific time frame. Our findings demonstrated that RIF is associated with inflammatory response and immune disorders, manifested by the production of pro-inflammatory factors and the imbalance of CD4^+^/CD8^+^ T cells. However, FZHY treatment restored the above results and showed a significant anti-fibrotic activity in UUO rat models. Mechanistically, FZHY may protect RIF by alleviating related inflammation and upregulating the ratio of CD4^+^/CD8^+^ T cells to improve the condition of “Healthy Qi Deficiency and Evil Qi Excess”.

SCR and BUN are markers of renal function parameters^17^. In the present study, FZHY reduced the levels of SCR and BUN on days 7, 14 and 21, suggesting that FZHY may have a protective effect on UUO induced RIF rats. Further, the effect was most significant on the 21st day, and there was no difference with positive control. TCM explains that rats with renal failure have syndromes of deficiency of spleen and kidney and internal resistance of turbidity and blood stasis, which is a typical “positive deficiency and evil syndrome”^14^. The serum CRP, TNF-α, IL-1, IL-6 and other microinflammatory indicators were used to evaluate the “excessive evil” situation. Numerous studies reveals that the continuous increase of inflammatory signals was closely associated with RIF, in which TNF-α, IL-1, IL-6 and CRP in renal tissue is a necessary condition for the progression and deterioration of RIF^18^8^20^. The data from the current study showed that FZHY significantly promoted the reduction of inflammatory factors including CRP, TNF-α, IL-1, and IL-6 in renal tissue on days 14 and 21. This further confirmed that FZHY can regulate the “evil Qi excess” in UUO induced RIF rats. However, FZHY failed to significantly reduce IL-1 levels on day 7. It’s not a contradiction, possibly due to the small sample size included or the fact that FZHY itself is truly insensitive to IL-1 in the early stage of renal fibrosis.

Immunosuppression is the main factor that determines the poor prognosis of patients with CKD, and its main feature is the immune response triggered by T lymphocytes, which exerts a critical effect in the initiation of renal fibrogenesis^21^. It has been reported that the changes of cellular immune indicators (CD4^+^ T cells, CD8^+^ T cells) are closely related to the condition of “positive deficiency syndrome” in the TCM^22^. Previous clinical studies demonstrated that the low ratio of CD4^+^/CD8^+^ in peripheral blood was closely related to poor outcome in liver fibrosis^23^ and lung fibrosis^24^. A previous study showed that the ratio of CD4^+^/CD8^+^ cells in patients with deficiency syndrome was significantly lower than that in normal subjects^25^. Similarly, we estimated the CD4^+^/CD8^+^ ratio in renal tissues, and the data showed that CD4^+^/CD8^+^ decreased significantly in the UUO group, but it was restored by FZHY treatment, suggesting that RIF is associated with the imbalance of CD4^+^/CD8^+^ ratio. These findings have also been found in previous studies of patients with CKD or ERSD^7^. Taken together, RIF has the macro and micro manifestations of “Healthy Qi Deficiency and Evil Qi Excess” according to TCM syndrome differentiation. FZHY prescription can improve the condition of “Healthy Qi Deficiency and Evil Qi Excess” and achieve therapeutic effect^14^.

Renal fibrosis is an important factor in the progression of renal disease to ESRD. Therefore, improving renal fibrosis is of great significance for the therapeutic intervention of renal failure. Increased inflammation and immunologic inadequacy promote the activation of renal fibroblasts, leading to the excessive deposition of extracellular matrix components (FN, LN, Col-I and Col-III), collagen formation, and, finally, the exacerbation of renal tissue fibrosis^26^-^28^. In the present study, AST-120 significantly reduced the levels of inflammatory indicators and fibrosis associated proteins in UUO model. Meanwhile, the findings of the present study demonstrated that FZHY inhibited kidney tissues fibrosis in UUO model *in vivo* and decreased the protein levels of fibrotic markers FN, LN, Col-I and Col-III. In all, the results indicated that FZHY can effectively improve renal fibrosis in UUO induced RIF model.

It has been established that AST-120 adsorbs indole, a precursor of indoxyl sulfate (IS), and reduces its eventual conversion to indoxyl sulfate in serum and urinary, thereby ameliorating interstitial fibrosis in CKD^29^. IS is able to activate NF-κB and TGF-β1/Smad3 signal in tubular cells and induces production of TNF-α by monocytes^30^-^32^, which are classical inflammation and/or fibrosis triggers. After UUO, IS accumulation is aggravated, but its suppression attenuated the progression of renal interstitial fibrosis^33^. Reduced CD4^+^/CD8^+^ ratio is associated with CKD, and the inverted CD4^+^/CD8^+^ ratio is a cause of ESRD^34^,^35^. AST-120 has been found by other researchers to significantly decrease CD8^+^ T cells (-33.9% for central memory T cells, and -42.6% for CD8^+^ naïve T cells), but it does not affect or slightly reduce CD4^+^ T cells (0 for T helper cell number, and -13.1% for early activated CD4^+^ T cells)^36^, which are consistent with our findings in this study. The reduced uremic toxins, such as indoxyl sulfate and p-cresyl sulfate, maybe involved in the phenomenon^37^-^40^. However, the exact mechanism of AST-120 exerting its anti-CKD effect still needs further study. Quite different from AST-120, FZHY doesn’t directly absorb uremic toxins like IS. Some of its main components, such as hederagenin, have been proven to have inhibitive effects on inflammation and fibrosis in renal after injury or disease^41^,^42^. In addition, although the mechanisms are different, our results showed a trend that the extended course of FZHY treatment may benefit more the CKD rats like AST-120 does. We deduce that increasing dose may also bring more positive results for CKD treatment. These findings suggest an alternative treatment for us to suppress inflammation and fibrosis in CKD in clinical apart from AST-120, which warrants further attention from the researchers in this field.

## Conclusion

The use of FZHY improves the condition of “Healthy Qi Deficiency and Evil Qi Excess” in UUO induced RIF rats, which is manifested as the facts that FZHY can reduce kidney tissue inflammation and induce the rebalance of CD4^+^/CD8^+^ ratio, and improve the UUO-induced renal fibrosis, providing a better understanding of anti-inflammatory and anti-fibrosis effect of FZHY in RIF.

## Supplementary

**Supplementary Table 1:** The list of core ingredients of FZHY in the treatment of chronic renal failure

FZHY composition	Core ingredients
Wine rhubarb	Torachrysone-8-O-beta-D-(6'-oxayl)-glucoside
	Emodin-1-O-beta-D-glucopyranoside
	Physciondiglucoside
Raw astragalus	Hederagenin
	Mairin
	Bifendate
*Rehmannia glutinosa*	Stigmasterol
	Sitosterol
Salvia	Isotanshinone II
	Digallate
	Salvilenone
Safflower	Flavoxanthin
	Baicalin
	Beta-carotene
Wine scutellaria	11,13-Eicosadienoic acid, methyl ester
	5,2'-Dihydroxy-6,7,8-trimethoxyflavone
	Epiberberine
Raw licorice	Glycyrin
	Licorice glycoside E
	18α-hydroxyglycyrrhetic acid
Wine leeches	Crocetin
	Nadroparin
	Ursolic Acid
Soil beetle	Aflatoxin B1
	Thymine
	Oleic acid

FZHY: FuZhengHuaYuJiangZhuTongLuo recipe.
